# Analysis of miRNAs and their target genes associated with mucosal damage caused by transport stress in the mallard duck intestine

**DOI:** 10.1371/journal.pone.0237699

**Published:** 2020-08-18

**Authors:** Hao Zhang, Fang Chen, Zhenhua Liang, Yan Wu, Jinsong Pi, Lixia Wang, Jinping Du, Jie Shen, Ailuan Pan, Yuejin Pu

**Affiliations:** 1 Institute of Animal Husbandry and Veterinary Sciences, Hubei Academy of Agricultural Sciences/Hubei Key Laboratory of Animal Embryo Engineering and Molecular Breeding, Wuhan, PR China; 2 Institute of Animal Husbandry and Veterinary Sciences, Wuhan Academy of Agricultural Sciences, Wuhan, PR China; Universidade Federal de Santa Maria, BRAZIL

## Abstract

Bowel health is an important factor for duck rearing that has been linked to feed uptake and growth and death rates. Because the regulatory networks associated with acute stress-mediated injury in the duck gastrointestinal tract have not clearly elucidated, we aimed to explore potential miRNA-mRNA pairs and their regulatory roles in oxidative stress injury caused by transport stress. Here, 1-day-old mallard ducklings from the same breeder flock were collected and transported for 8 h, whereas the control group was not being transported. Various parameters reflecting oxidative stress and the tissue appearance of the intestine were assessed. The data showed that the plasma T-AOC and SOD concentrations were decreased in the transported ducklings. The intestine of the transported ducklings also displayed significant damage. High-throughput sequencing of the intestine revealed 44 differentially expressed miRNAs and 75 differentially expressed genes, which constituted 344 miRNA-mRNA pairs. KEGG pathway analysis revealed that the metabolic, FoxO signaling, influenza A and TGF-β signaling pathways were mainly involved in the mechanism underlying the induction of intestinal damage induced by simulated transport stress in ducks. A miRNA-mRNA pair, miR-217-5p/*CHRDL1*, was selected to validate the miRNA-mRNA negative relationship, and the results showed that miR-217-5p could influence *CHRDL1* expression. This study provides new useful information for future research on the regulatory network associated with mucosal damage in the duck intestine.

## Introduction

The negative effect caused by environmental stress has been increasingly recognized and studied. Transportation stress is complicated by various physical challenges, including fasting, dehydration, motion, crowding and high temperature [1]. An acute stress model can be established using transportation, which induces a marked increase in the level of reactive oxygen species (ROS) [2, 3]. The interaction between high concentrations of ROS and cell membrane constituents leads to lipid peroxidation, membrane disintegration and cell damage [4]. Studies have shown that oxidative stress acts as a stimulus for stress-induced intestinal mucosal injury [5]. The intestinal tract of ducks possesses a large surface area, allows the exchange of nutrients into the systemic circulation, and prevents penetration of toxic compounds [6–8], and maintenance of the integrity of the intestinal mucosa is important [9, 10].

MicroRNAs (miRNAs) are endogenous, non-coding short RNAs that can hybridize to the 3’ untranslated region of the mRNAs of target genes to direct their posttranscriptional regulation [11, 12]. A large number of miRNAs have been identified, and a single miRNA can inhibit the production of hundreds of mRNAs of protein-coding genes in a relatively mild manner [13]. Changes in miRNA expression levels has been associated with many biological processes, such as apoptosis and cell-cycle progression, inflammation, cancer, and tissue differentiation and regeneration [14–16]. Moreover, previous studies have confirmed that miRNAs are also involved in animal responses to oxidative stress [17, 18]. For example, oxidative stress increases the levels of miR-383, miR-34a and miR-21 and downregulates the expression of other miRNA families, including miR-106a and miR-15b [19]. In addition, in RPE cells, miR-383 is elevated in response to high glucose-induced oxidative stress and represses PRDX3 gene expression [20]. *PRDX3* is considered a key antioxidant in oxidative stress, and PRDX3 interacts with the MAPK signal pathway [21]. Although the biological significance of many miRNAs related to oxidative stress has been elucidated and functional studies have implicated their regulatory actions, previous studies have revealed that miRNAs are differentially expressed in the stress response processes in mammals [22]. The production performance of ducks, which are a type of timid poultry, can be reduced by transport stress, and the stress response in the duck intestine might be somewhat different. Furthermore, the available information regarding the dysregulated expression of miRNAs associated with intestinal mucosal damage induced by transport in ducks is scarce.

To investigate the effect of transport stress on the duck intestine, this study placed 1-day-old ducklings in duck transport vehicles and drove them for 8 hours, and the physical conditions of the ducks and various biochemical indicators were then tested. The morphological changes in the duck intestine were examined using a light microscope. To further understand the correlation between the expression of miRNAs and their target genes in the intestine affected by transportation, we profiled the expression of miRNAs and mRNAs in the intestine of newborn ducks after 8 h of transport and that of newborn ducks that were not transported. We further confirmed the expression of miRNAs and their predicted target genes in the intestine using qRT-PCR and various methods to comprehensively assess their potential biological functions.

## Materials and methods

### Animals and ethics statement

The methods used in this study were based on the Guide to Laboratory Animals developed by the Ministry of Science and Technology. All the procedures involving animal subjects were approved by the Animal Ethics Committee of the Hubei Academy of Agricultural Sciences.

### Treatments and sampling

All birds were hatched at Hubei LiHu health poultry egg co., LTD. In this study, two groups (n = 10) of 1-day-old ducklings with the same background were established: the treatment group (TG) and the control group (CG). The ducklings that comprised the control group were transported directly from the hatchery to the barn, which was a distance of approximately 100 m, and these ducklings were considered non-transported ducklings. The temperature of the barn was 25~27°C. The control group ducklings were not provided feed and water, and the density was 50 per square meter. The ducklings that comprised the treatment group were transported under commercial conditions using an air-conditioned semi-trailer tractor for 8 hours, and during the test, the temperature was between 24 and 29 centigrade. In the process of transportation, ducklings are packed in ducklings transport boxes, each box contains 20 ducklings. The dimension of the boxes is 17cm high, 25 cm wide and 40 cm long. As with the control group, the treatment group ducklings were not provided feed and water. After 8 hours of treatment, a contact thermometer and an electronic balance were used to determine the body surface temperature and weight, respectively. Six ducks were randomly selected from each group euthanatized by jugular venesection. The electrical stunning equipment was used to stun the ducklings before blood letting them to death. Their duodenum was divided into two sections: (1) a 1-cm-long section was fixed with 10% neutral formalin for paraffin embedding and (2) a 1-cm long section was chopped, dispensed into a sample tube, frozen in liquid nitrogen, and then stored at -70°C. Blood samples (4ml per sample) were collected from the carotid artery and centrifuged at 3000 g for 10 min, and the serum was stored at -20°C until use.

### Analysis of serum biochemistry variables

Serum corticosteroids (CORTs) were measured using an ELISA kit for ducks according to the manufacturer's instructions (Mlbio, Shanghai, China). The levels of total superoxide dismutase (T-SOD), malondialdehyde (MDA) and total antioxidants (T-AOCs) were determined using an appropriate assay kit according to the manufacturer's instructions (Jiancheng Bioengineering Institute, Nanjing, China).

### Morphological examination

Formalin-fixed samples were paraffin-embedded and laterally sliced to a thickness of 5 μm. After dewaxing and dehydration, the intestine sections were stained with hematoxylin and eosin (HE). The morphology of the intestinal mucosa was observed using a Nikon E200 microscope (Nikon, Tokyo, Japan) and analyzed using an image analysis system.

### RNA extraction

Total RNA was extracted from the intestine of each duck using TRIzol (Invitrogen, CA, USA) and then treated with RQ1 DNase (Promega, USA) to remove DNA. The RNA quality and quantity were assessed using a NanoDrop 1000 spectrophotometer (Thermo Fisher Scientific, Waltham, MA, USA). The RNA concentration was measured using a Qubit® RNA Assay Kit with a Qubit®2.0 fluorometer (Life Technologies, CA, USA). The RNA integrity was assessed using an Agilent Bioanalyzer 2100 system (Agilent Technologies, CA, USA).

### RNA sequencing

Three intestine samples from each group were randomly selected for sequencing. For each sample, a build sequence library was generated using 3 μg of RNA using the NEB Next Ultra RNA Library Preparation Kit (Illumina, San Diego, CA, USA). The mRNA was purified using magnetic beads with poly-T oligonucleotides and fragmented into small pieces, and the cleaved RNA fragments were reverse transcribed into cDNA. Novogene Inc. (Tianjin, China) performed single-ended sequencing of each library using the HiSeq 2000 platform. Using the Tophat program, we mapped all clean reads obtained from the RNA sequencing analysis to the Anas platyrhynchos genome based on three mismatches, and the annotation data were downloaded from www.ncbi.nlm.nih.gov/genome. To compare the mRNA abundances, the read count for each mRNA was normalized to RPKMs (genes per kilobase read per million gene reads). The differentially expressed genes in both groups were identified based on RPKM ≥ 100 and P value < 0.01.

### Small RNA sequencing and computational analysis

According to the instructions, 3 μg of total RNA was used for the preparation of a small RNA library. High-throughput sequencing was performed using Novoligene's Illumina HiSeq 2000 platform (Tianjin, China). Purified small RNA libraries were quantified using a Qubit fluorometer (Invitrogen, Shanghai, China), and cluster generation and 36-nucleotide single-ended sequencing analysis were performed using Illumina GAIIx (Illumina, San Diego, CA, USA) according to the manufacturer's instructions. After removing the adapter, low-quality and contaminant reads, the clean reads were aligned with the duck genome. The RfamA reads aligned to a mismatch and with between 19 and 25 nucleotides were inputted to miRDeep for prediction of a new miRNA locus. The miRNA expression levels were normalized based on the number of fragments per kilobase (FPKMs) per transcript. The differentially expressed miRNAs in both groups were identified based on FPKM ≥ 100.00 and P < 0.01.

### miRNA-mRNA correlation and pathway analysis

The miRNAs and genes were significantly downregulated in the intestinal tissue of the TG compared with the CG. The Pearson-related expression coefficients for the mRNA-miRNA associations were calculated using R. For each potential interaction, which is defined as a miRNA-mRNA pair with a Pearson correlation coefficient < 0.5, we determined whether the mRNA was predicted or confirmed through a search of the miRNAs in the miRWalk (version 1.0) and miRTarBase databases (release 4.5). In addition, we used GeneTrail to analyze the overexpression of all negatively related genes to determine which KEGG pathways are significantly affected by the dysregulation of gene expression during transport. A miRNA-mRNA interaction network was constructed using Cytoscape 3.5.1 software. The target gene was then subjected to GO term and KEGG pathway analyses. Subsequently, potential target genes for the differentially expressed miRNAs were predicted by matching the miRNA 3'-UTR sequences and assessing their energy stability using the Miranda algorithm.

### Validation of miRNAs and mRNA by qRT-PCR

Total miRNAs from the gut were extracted using the miRcute miRNA isolation kit (TIANGEN, China), and total RNA from the intestine was extracted using the TRIzol reagent (Takara, Osaka, Japan) according to the manufacturer's recommended protocol and treated with recombinant DNase I (Takara). The reverse transcription of miRNA into cDNA was performed using the gDNA Eraser PrimeScript RT kit (Takara, Japan) and specific stem-loop primers. S3 Table lists the primers used in the qRT-PCR analysis. qRT-PCR was performed with an ABI 7300 system (Applied Biosystems, CA, USA) using the THUNDERBIRD SYBR qPCR Mix (Japan TOYOBO). U6 and β-actin were used as reference genes, and the relative expression levels of miRNA and mRNA were calculated using the 2^-△△Ct^ method.

### Dual-luciferase reporter assay

According to the manufacturer's instructions, the 3'-UTR of the miRNA-binding site containing the target gene was amplified, and the pmirGLO dual luciferase miRNA target was recombined into an expression vector. The miRNA mimic or negative control (GenePhama, China) and the validated vector plasmid were transfected into primary duck intestinal epithelial cells using Lipofectamine 3000 (Life Technologies, USA). Twenty-four hours after transfection, the luciferase activity was detected using a dual luciferase reporter assay system (Promega, USA).

### Cell culture and transfection

The main duck intestinal epithelial cells were isolated from 26-day-old duck embryos through enzymatic digestion [20]. The cells were cultured in 5% FBS in DMEM/F12 medium (Gibco, USA) and incubated at a density of 3 × 10^5^/cm^2^ on a 24-well cell culture dish (Sangon, China) at 37°C in the presence of 5% CO_2_. The medium was changed every 36 hours. After 36 h of culture, the cells were divided into three groups: blank control group (BC), negative control group (NC), and miR-217-5p mock group. Transfection was performed using Lipofectamine 2000 (Invitrogen, USA) according to the manufacturer's instructions. Forty-eight hours after transfection, the cells were collected for detection of the expression level of *CHRDL1*.

### Statistical analysis

The differences between two groups were analyzed using SPSS software based on a two-tailed Student’s *t*-test. Differences were considered significant if *P* < 0.05. All the data are expressed as the means ± standard deviations (S.Ds.).

## Results

### Body weight, body surface temperature, serum biochemical indicator concentration and morphological analysis

**Fig 1:**
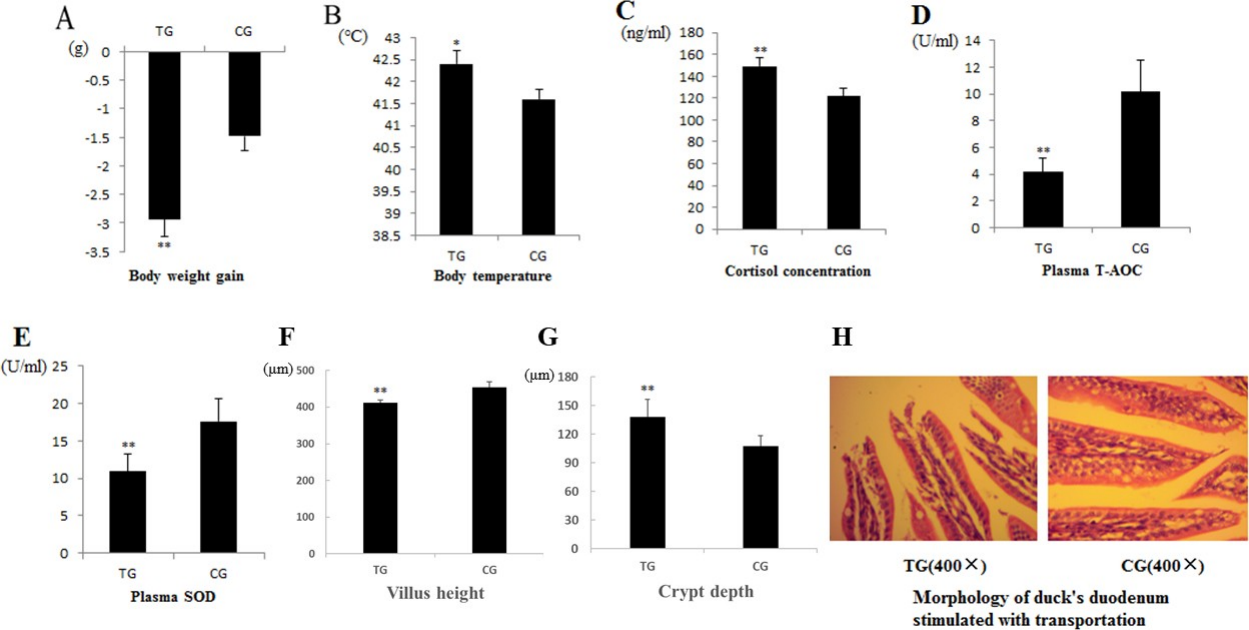
Effects of transport treatment. Comparisons of the (A) body weight, (B) body temperature, (C) plasma CORT concentration, (D) plasma T-AOC concentration, (E) plasma SOD concentration, (F) villus height, (G) crypt depth and (H) histological observations (400×) of the intestine between the transported and control ducks. * indicates P < 0.05, ** indicates P < 0.01.

After 8 h of transport, the body weights of the ducks were reduced, and their body surface temperatures were significantly elevated. The ducks in the TG experienced oxidative stress induced by transport. The serum CORT concentration in the transported ducks was significantly higher than that in the control ducks, and the SOD and T-AOC concentrations in the TG were significantly decreased compared with those in the CG. In addition, the transport treatment caused severe damage to the small intestine of the ducks, mainly manifested as mechanical damage at the top of intestinal villis, the villus height decreased and the crypt depth increased (P<0.01) (Fig 1).

### Deregulated mRNA expression in the TG compared with the CG

**Fig 2:**
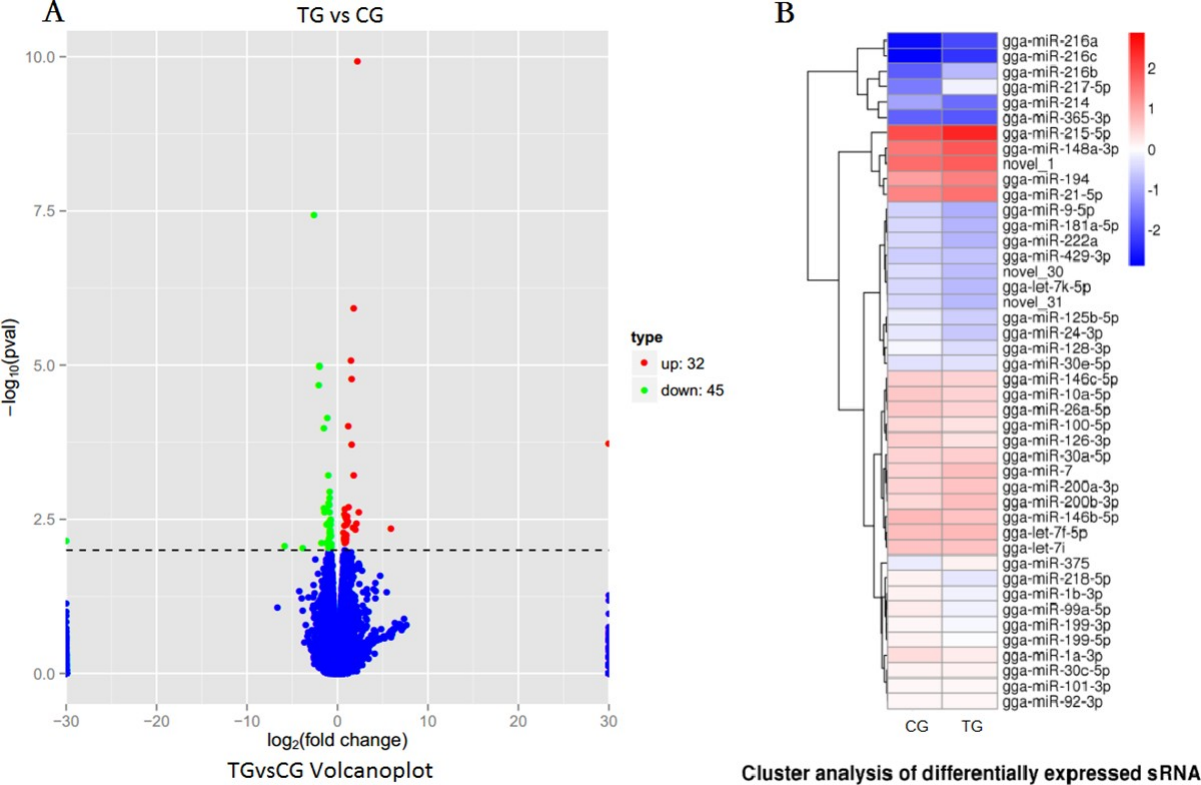
Heatmap of differentially expressed genes and differentially expressed miRNAs in the CG and TG intestine. (A) Heatmap of differentially expressed genes in the TG compared with the CG. (B) Differentially expressed miRNAs in the TG compared with the CG.

The intestine transcriptome was analyzed using an Illumina platform, and the gene expression level was represented by the expected number of fragments per kilobase of transcript sequence per million base pairs sequenced (FPKM), which is a normalized, quantitative metric that considers the effects of gene sequencing depth and the length of the reads used for gene expression analysis (Trapnell, Cole, et al., 2010). The FPKM value for each gene was calculated using HTSeq software, and a frequency analysis of the number of genes within specific FPKM classes was performed. The differentially expressed tags between the two groups were identified using edgeR software. In total, 77 differentially expressed genes (DEGs) were identified among the two groups, and these included and 32 upregulated and 45 downregulated genes in the TG compared with the CG (Fig 2A). All of the DGEs are listed in S1 Table.

### miRNA expression profiles in the duck intestine

The sequencing analysis yielded information on the total of miRNAs in all intestine samples. Furthermore, the statistical results indicated that 44 miRNAs were differentially expressed in the TG compared with the CG intestine, and among these, 19 and 25 miRNAs were upregulated and down-regulated in the TG. The differential expression levels of the 44 miRNAs in the CG and TG are presented in Fig 2B. A comparison with the known miRNAs in the miRBase database revealed that 41 of the 44 differentially expressed miRNAs are known, and the other three are thus novel miRNAs (the mature sequences are displayed in S2 Table).

### Relationship between of miRNA and mRNA expression and functional analysis

**Fig 3:**
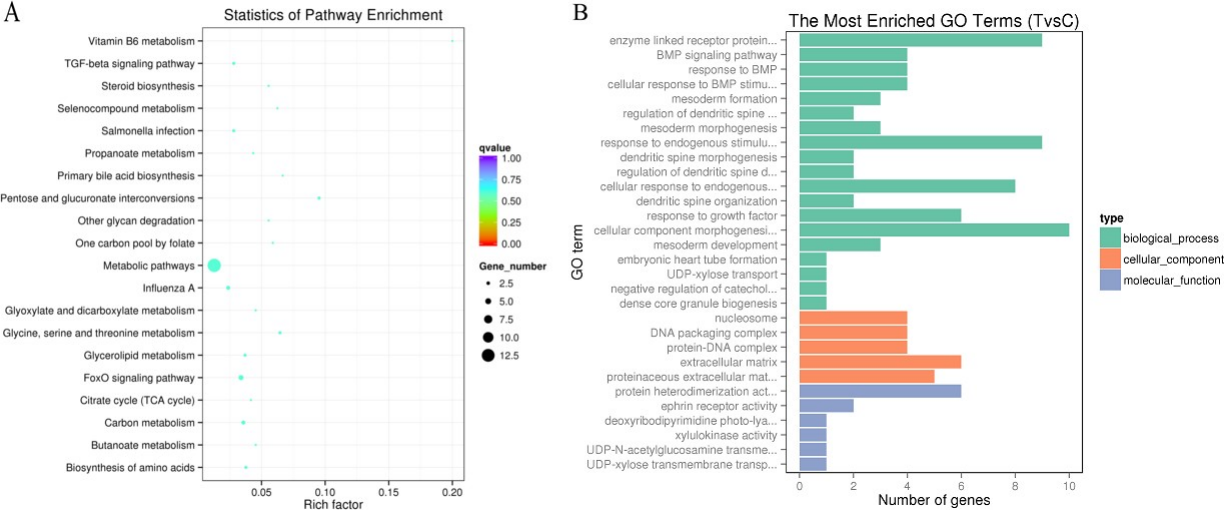
Significantly enriched pathways involved in intestinal damage. (A) KEGG pathway analysis of the putative target genes of the differentially expressed miRNAs. (B) Top enriched GO term categories of the putative target genes.

**Fig 4:**
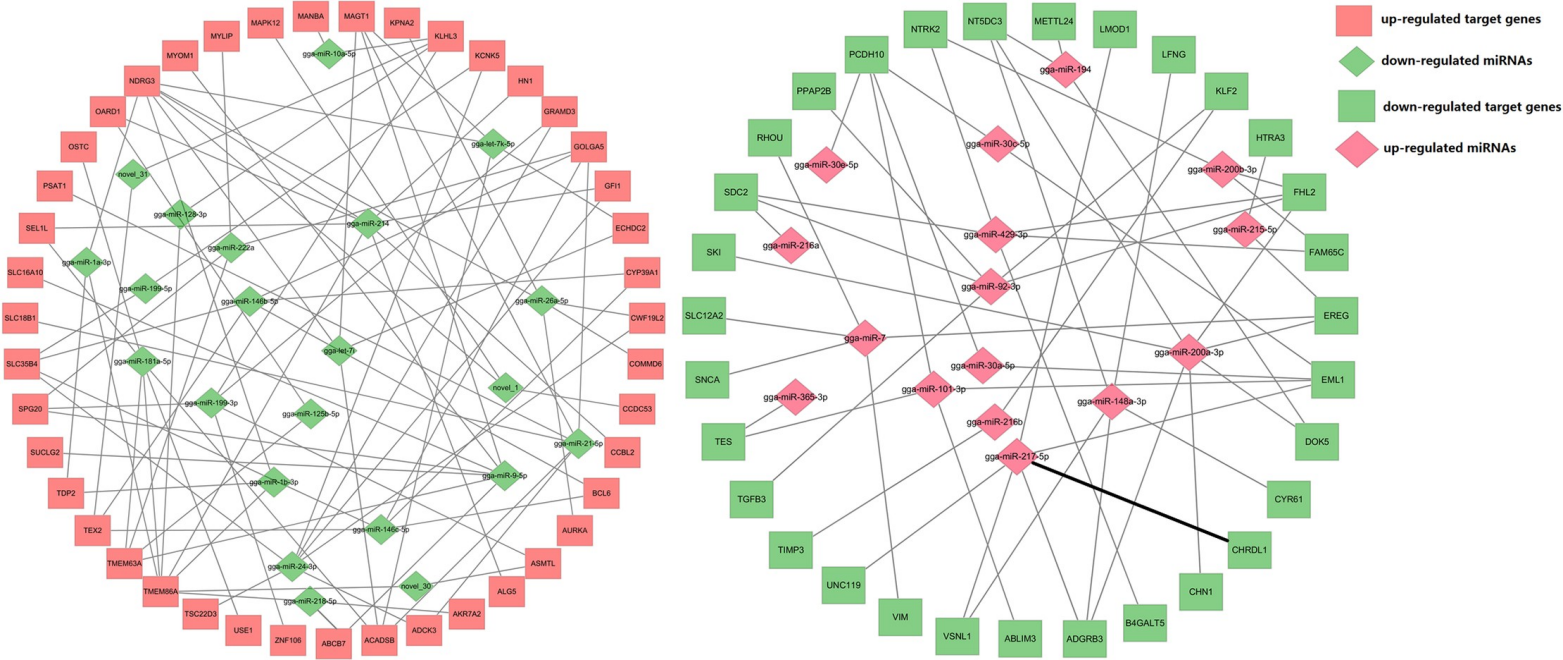
Regulatory networks of the differentially expressed miRNAs and their putative genes involved in intestinal damage.

To further understand the relationship between miRNA and mRNA changes and to specifically identify potentially relevant miRNA-mRNA target interactions, we computed the Pearson correlation coefficient for each miRNA/mRNA pair corresponding to the 44 deregulated miRNAs and 77 deregulated mRNAs. This analysis identified 344 miRNA-mRNA pairs with inversely correlated expression, and these potential interactions are included in miRWalk as predicted. To identify the biological functions of miRNAs in ducks, we performed a KEGG pathway analysis of the differentially expressed potentially miRNA-regulated genes. The results revealed several main groups of KEGG pathways: (i) metabolic pathway; (ii) FoxO signaling pathway; (iii) influenza A pathway and (iv) TGF-β signaling pathways (Fig 3). An miRNA-mRNA interaction network was constructed using Cytoscape 3.5.1 software (Fig 4).

### Validation of selected miRNA and mRNA by qRT-PCR

**Fig 5:**
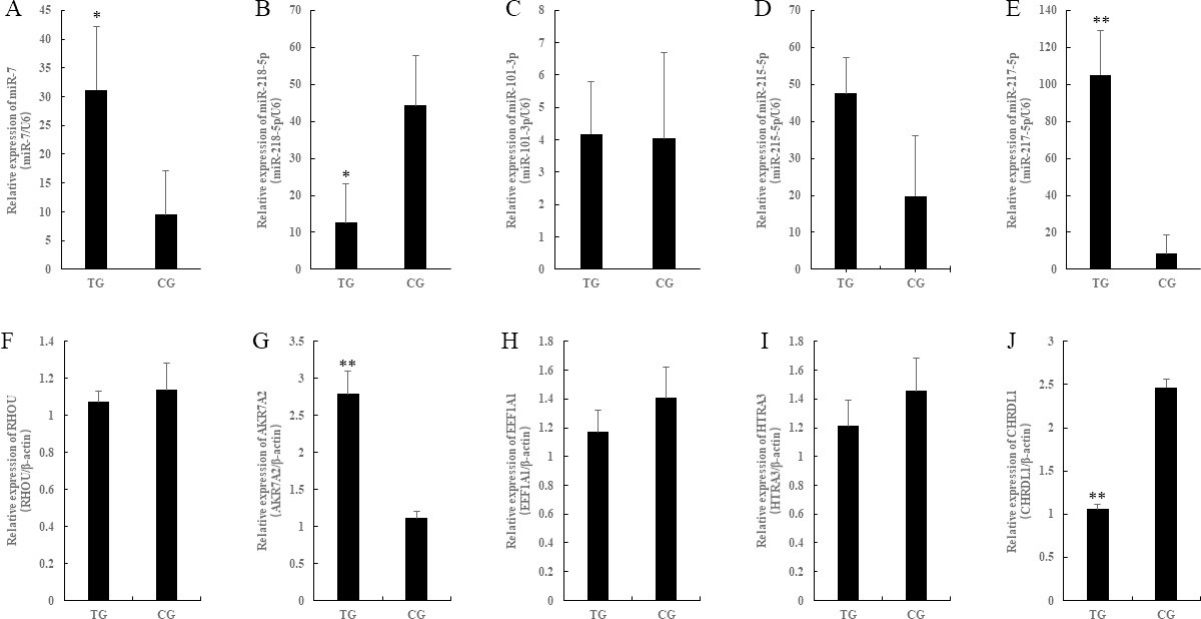
Validation of selected miRNAs and Mrna by qRT-PCR. *Means P < 0.05, **means P < 0.01.

We used qRT-PCR technology to confirmed 5 differentially expressed miRNAs and 5 differentially expressed genes (Fig 5) which obtained by high-throughput sequencing. The result shows that the fold-change was differential between qRT-PCR and high-throughput sequencing. However, the variation trend was consistent between the two detection methods.

### Validation of the negative regulation of miRNA/mRNA pairs

**Fig 6:**
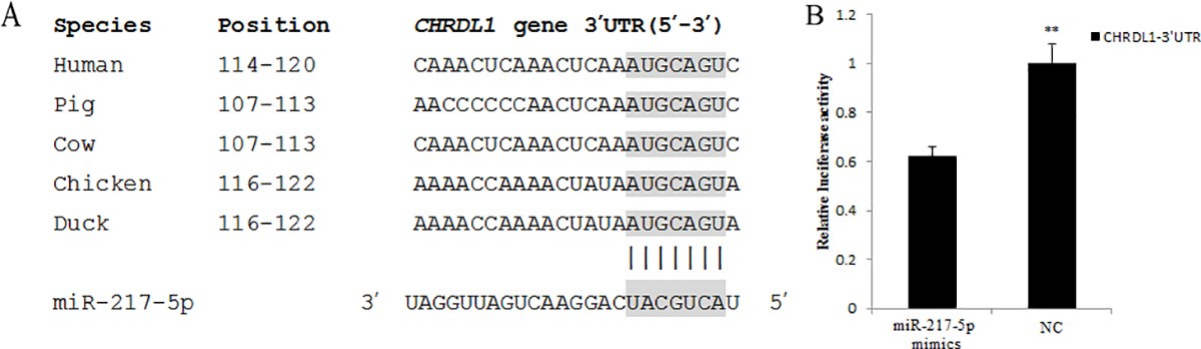
Validation of a select miRNA and a select target gene by qRT-PCR. (A) Binding sites for miR-217-5p in the *CHRDL1* 3’UTR of different species. (B) Luciferase activity assay of the recombined dual-luciferase reporter vectors with the 3’UTR of *CHRDL1* co-transfected with miR-217-5p mimics or NC. ** indicates *P* < 0.01.

Relative expression of miR-217-5p was increased 9.5-fold after transport. The high-throughput sequencing and qRT-PCR analyses showed that miR-217-5p was differentially expressed. Among the genes that are potentially regulated by miR-217-5p, Chordin-Like 1 (*CHRDL1*) was chosen for qRT-PCR validation and dual-luciferase reporter system analysis. The recombined reporter vectors with the 3’-UTRs of CHRDL1 were co-transfected into duck intestine epithelial cells (IECs) with miR-217-5p mimics. Transfections with and without negative mimics were performed as negative controls (NCs) (Fig 6). Six replicates of each transfection were performed.

## Discussion

Due to the timid and skittish behavioral traits of ducks, adverse environmental conditions induce acute stress, which could lead to severe problems in duck production and thus large economic losses every year. Our study of the body surface temperatures, body weight, serum CORT concentration and histology of the intestine showed that the ducks experienced obvious stress reactions after 8 hours of transport, and the intestine of the ducks exhibited mucosal injury. In addition, after the transportation, the ducklings in the TG showed shortness of breath, feathers wetted, and a certain degree of dehydration. Physical abnormalities have been observed in mammals in response to transport stress [23]. In 1-day-old broilers, 10 hours of transport could decrease production performance during the first 2 weeks [24]. Glucocorticoids are critical for environmental adaptation, and increased serum CORT levels reveal the occurrence of a stress response [25]. The above phenotype might be caused by sympathetic nerve excitement during stress. Numerous published studies have shown that the integrity of the intestinal epithelia and the villi height are decreased in pigs and rats that have been transported for a long distance [26, 27]. Normally, after more than a century of hybridization and improvement, chicken has a stronger adaptability to stress. The systematic breeding of ducks is low, and the test results are based on animal nature, it can better reflect the stress response mechanism. Therefore, the duck intestine can be a good model for investigating the mechanism of gastrointestinal reactions in poultry during exposure to stress.

Oxidative stress, which is caused in part by adverse environmental conditions, is another important characteristic associated with transport. To date, many researchers have focused on oxidative stress in mammals, such as ischemia reperfusion injury [28] and heat stress [29]. The occurrence of oxidative stress occurs increases the blood serum levels of ROS and thus induces lipid peroxidation [30, 31]. ROS are a potential factor triggering intestinal injury and dysfunction in animals under stress [32], and the detrimental effects of ROS in the injury process have been established [33]. Excessive levels of ROS damage cytoskeletal proteins, disrupt the intestinal barrier, and increase gut permeability [34]. In this study, the serum T-AOC and SOD levels were significantly higher in the experimental ducks than in the control ducks. Related studies have shown that long-distance transport leads to increase in the ROS concentrations in mammals and thus changes in the activities of antioxidant enzymes [30, 35]. Based on these findings, we deduced that oxidative stress induces intestinal mucosal injury in transported ducks.

The duodenum is an important node of the hepatic and intestinal circulation, receiving both gastric juice, pancreatic juice and bile. In addition, relevant study shows that the duodenum and jejunum is stress sensitive parts of the small intestine in the process of stress response, so we chose the duodenum as the target to study the expression of miRNAs and genes. To the best of our knowledge, this study constitutes the first attempt to simultaneously analyze both the mRNA and miRNA expression profiles in the duck intestine mucosa after transport-induced damage. We discovered 77 DEGs in the TG, and the RNA-seq analysis revealed that the significantly altered genes were mainly related to the enzyme-linked receptor protein signaling pathway (GO:0007167), cellular component morphogenesis (GO:0032989), the cellular response to endogenous stimulus (GO:0071495) and the cellular response to BMP stimulus (GO:0071773). Furthermore, a KEGG pathway analysis revealed that the metabolic, FoxO signaling, influenza A and TGF-β signaling pathways were the main pathways involved in the mechanism underlying the induction of damage in the duck intestine by simulated transport stress. The differential expression of these mRNAs could be due to miRNA regulation or other mechanisms, which need to be further researched.

miRNAs are endogenous small noncoding RNAs that direct the posttranscriptional regulation of gene expression by inhibiting the translation and/or inducing the degradation of target mRNAs. Many studies have shown that numerous miRNAs are involved in the regulation of the stress response [36]. The miRNAs involved in stress-induced intestinal mucosal injury in ducks remain to be elucidated. In this study, we found that 41 conserved miRNAs and three novel miRNAs were differentially expressed in the duck intestine after transport, and the identification of the functional miRNAs involved in the induction of injury to the intestinal mucosa is important. Despite the large amount of differentially expressed miRNA and DEG were obtained through High throughput sequencing, this result was mainly caused by the comprehensive factor of long-distance transportation. However, the transportation process includes various environmental stimuli such as bumps, high density, and heat stress. It remains unclear which factor is the main cause of the transcript differential expression, and it is worthy to be studied further through a single modeling experiment, to clarify the chief cause of transportation stress in ducklings, and to provide an effective response way.

Some dysregulated miRNAs identified in this study were consistent with the results obtained in other studies. miR-217 has been recognized as a tumor suppressor that is downregulated in various types of cancer. Recent studies have shown that miRNA-217 could inhibit cell proliferation in acute myeloid leukemia, hepatocellular carcinoma and breast cancer [37–39]. However, in glioblastoma tissues and cells, the expression of miR-217 could promote the proliferation and invasion of glioblastoma [40]. miRNA-217 also plays an important role in the process related to the induction and repair of tissue injury. The inhibition of miR-217 can protectively antagonize high glucose-induced podocyte damage [41]. However, its expression level and mechanism of action in the small intestine have not been reported and thus remain to be further investigated.

To understand the biological function of differentially expressed miRNAs, we predicted their target genes and classified the identified genes according to their pathways through a KEGG pathway analysis. The *CHRDL1* gene plays key roles in TGF-β pathways, which might lead to the development of mucosal injury in the duck intestine, and was selected for validation. A sequence analysis between miR-217-5p seed regions and target 3’-UTRs was performed using TargetScan (http://www.targetscan.org/). The relative luciferase activity of recombined dual-luciferase reporter vectors with the 3’-UTR of *CHRDL1* was significantly decreased by miR-217-5p mimics. Chordin is a bone morphogenetic protein (BMP) inhibitor that has been identified as a factor involved Xenopus embryo dorsalization and has also been found in various microbes [42]. *CHRDL1*, which shows significant homology to chordin, was isolated from mouse bone marrow stromal cells [43]. However, due to the absence of an anti-CHRDL1 antibody suitable for ducks, the effects of miRNAs on the protein expression levels of their target genes was not measured. These results indicate that miR-217-5p might be involved in the regulation of stress-induced intestinal mucosal injury through the regulation of *CHRDL1* in the intestine. In addition, BMPs are extractable pluripotent cytokines with specific functions in the regulation of the extracellular matrix and extracellular space [44, 45]. Therefore, subsequent studies will focus on the function of the predictedmir-217-5p/*CHRDL1*/BMP signaling pathway.

## Conclusions

The results of the present study showed that transport can cause intestinal mucosa injury in ducks and that this effect might be related to oxidative stress. We constructed a miRNA library of the duck intestine and examined the expression of miRNAs and mRNA by high-throughput sequencing. We found that the miR-217-5p/CHRDL1 pair showed significant expression differences in the intestine of the transported compared with the non-transported ducks and might thus play an important role in oxidative stress-induced injury.

## Supporting information

S1 Table Differentially expressed known and novel mRNAs. (DOCX)

S2 Table Differentially expressed known and novel miRNAs. (DOCX)

S3 Table Detailed information of primers. (DOCX)

S1 File (DOCX)
