# Supplementary material for: Analysis of miRNAs and their target genes associated with mucosal damage caused by transport stress in the mallard duck intestine
Source: PLoS One. 2020 Aug 18;15(8):e0237699. doi: 10.1371/journal.pone.0237699 (PMC7437463; doi:10.1371/journal.pone.0237699)
Supplement: S1 Table — (DOCX) [file pone.0237699.s001.docx]

**Table S1. Differentially expressed known and novel mRNAs.**

| Gene_id | Gene_name | Expression in the TG | Expression in the CG | Fold change |
| --- | --- | --- | --- | --- |
| ENSAPLG00000008686 | Novel gene | 21.98 | 318.31 | 0.07 |
| Novel01602 | Novel01602 | 16.88 | 101.81 | 0.17 |
| ENSAPLG00000011263 | NR4A3 | 26.92 | 114.39 | 0.24 |
| Novel01300 | Novel01300 | 1620.32 | 6430.10 | 0.25 |
| ENSAPLG00000012948 | HSPB7 | 49.85 | 140.68 | 0.35 |
| ENSAPLG00000010520 | FOXO6 | 45.13 | 104.80 | 0.43 |
| ENSAPLG00000001097 | TMEM100 | 98.96 | 224.89 | 0.44 |
| ENSAPLG00000011890 | LAMA2 | 217.40 | 473.44 | 0.46 |
| ENSAPLG00000013276 | EPHA2 | 339.17 | 691.64 | 0.49 |
| ENSAPLG00000009602 | KLF2 | 378.52 | 752.44 | 0.50 |
| Novel00888 | Novel00888 | 61.70 | 122.31 | 0.50 |
| ENSAPLG00000005130 | TC2N | 91.13 | 179.16 | 0.51 |
| ENSAPLG00000015814 | NLGN3 | 78.90 | 154.97 | 0.51 |
| ENSAPLG00000001689 | GPR20 | 66.85 | 128.09 | 0.52 |
| ENSAPLG00000008292 | ARSJ | 59.90 | 114.31 | 0.52 |
| Novel01019 | Novel01019 | 1083.40 | 2060.03 | 0.53 |
| ENSAPLG00000004392 | RHOU | 449.75 | 845.55 | 0.53 |
| ENSAPLG00000007917 | PXDNL | 79.51 | 146.02 | 0.55 |
| ENSAPLG00000006664 | PMP22 | 206.93 | 378.87 | 0.55 |
| ENSAPLG00000008673 | ADAMTS1 | 546.50 | 997.95 | 0.55 |
| ENSAPLG00000011879 | Novel gene | 117.08 | 213.23 | 0.55 |
| ENSAPLG00000009611 | EPHA4 | 210.45 | 380.44 | 0.55 |
| ENSAPLG00000012721 | RPAIN | 119.02 | 214.21 | 0.56 |
| ENSAPLG00000011156 | CFTR | 949.52 | 1708.30 | 0.56 |
| ENSAPLG00000002018 | EGR1 | 203.96 | 360.58 | 0.57 |
| ENSAPLG00000002582 | WISP1 | 251.96 | 439.89 | 0.57 |
| ENSAPLG00000015054 | Novel gene | 183.91 | 320.52 | 0.57 |
| ENSAPLG00000012542 | BAIAP2 | 153.47 | 266.50 | 0.58 |
| ENSAPLG00000012982 | EREG | 578.23 | 1002.81 | 0.58 |
| ENSAPLG00000006628 | CADM3 | 311.14 | 532.80 | 0.58 |
| ENSAPLG00000010991 | HTRA3 | 414.94 | 706.08 | 0.59 |
| ENSAPLG00000009922 | PDLIM3 | 815.27 | 1369.15 | 0.60 |
| ENSAPLG00000014103 | NDNF | 468.80 | 786.87 | 0.60 |
| ENSAPLG00000011574 | CHRDL1 | 589.96 | 965.79 | 0.61 |
| ENSAPLG00000007523 | FBN3 | 597.66 | 970.18 | 0.62 |
| ENSAPLG00000014027 | FBLN2 | 370.14 | 593.74 | 0.62 |
| ENSAPLG00000012868 | COL4A5 | 3011.08 | 4802.53 | 0.63 |
| ENSAPLG00000009162 | CHGA | 817.71 | 501.86 | 1.63 |
| ENSAPLG00000015129 | ACADSB | 1520.09 | 895.46 | 1.70 |
| ENSAPLG00000007665 | MACC1 | 484.82 | 283.49 | 1.71 |
| ENSAPLG00000012716 | XYLB | 584.43 | 338.03 | 1.73 |
| ENSAPLG00000004270 | Novel gene | 444.33 | 252.71 | 1.76 |
| ENSAPLG00000005885 | SLC35B4 | 806.53 | 454.98 | 1.77 |
| ENSAPLG00000002315 | Novel gene | 188.04 | 105.04 | 1.79 |
| ENSAPLG00000001835 | HSD17B2 | 159.42 | 84.36 | 1.89 |
| ENSAPLG00000004975 | Novel gene | 198.80 | 104.69 | 1.90 |
| ENSAPLG00000011759 | KCNJ15 | 189.64 | 98.96 | 1.92 |
| ENSAPLG00000010648 | MYOM1 | 100.99 | 51.18 | 1.97 |
| ENSAPLG00000003172 | Novel gene | 231.72 | 115.92 | 2.00 |
| ENSAPLG00000002269 | Novel gene | 125.56 | 62.56 | 2.01 |
| ENSAPLG00000002263 | HIST2H2AA4 | 149.14 | 74.01 | 2.02 |
| ENSAPLG00000008850 | Novel gene | 151.50 | 71.46 | 2.12 |
| ENSAPLG00000001623 | Novel gene | 276.24 | 130.11 | 2.12 |
| Novel00683 | Novel00683 | 173.20 | 76.13 | 2.28 |
| ENSAPLG00000013874 | LAPTM4B | 218.93 | 94.48 | 2.32 |
| Novel01859 | Novel01859 | 257.87 | 89.21 | 2.89 |
| ENSAPLG00000010001 | Novel gene | 532.44 | 178.21 | 2.99 |
| ENSAPLG00000005088 | OSGIN1 | 1369.88 | 454.92 | 3.01 |
| ENSAPLG00000011369 | KLB | 411.00 | 121.60 | 3.38 |
| ENSAPLG00000004623 | AQP7 | 141.55 | 40.53 | 3.49 |
| Novel00840 | Novel00840 | 101.25 | 28.58 | 3.54 |
| ENSAPLG00000015887 | Novel gene | 278.08 | 59.22 | 4.70 |

The differentially expressed mRNAs were identified based on FPKM ≥ 100.00 in either of the two groups, fold change ≥ 1.50 or ≤ 0.67, and P < 0.01.
