# Supplementary material for: Analysis of miRNAs and their target genes associated with mucosal damage caused by transport stress in the mallard duck intestine
Source: PLoS One. 2020 Aug 18;15(8):e0237699. doi: 10.1371/journal.pone.0237699 (PMC7437463; doi:10.1371/journal.pone.0237699)
Supplement: S2 Table — (DOCX) [file pone.0237699.s002.docx]

**Table S2. Differentially expressed known and novel miRNAs.**

| miRNA | Expression in the TG | Expression in the CG | Fold change | Reference |
| --- | --- | --- | --- | --- |
| apla-miR-218-5p | 1557.81 | 2917.23 | 0.53 | gga |
| apla-miR-126-3p | 5216.24 | 9277.27 | 0.56 | gga |
| apla-miR-99a-5p | 1936.86 | 3348.50 | 0.58 | gga |
| apla-miR-214 | 87.57 | 151.33 | 0.58 | gga |
| apla-miR-146b-5p | 9657.96 | 15828.76 | 0.61 | gga |
| novel_1 | 108192.90 | 166473.71 | 0.65 | gga |
| apla-miR-1b-3p | 1932.75 | 2864.09 | 0.68 | gga |
| apla-miR-24-3p | 711.59 | 1040.27 | 0.68 | gga |
| apla-miR-222a | 454.48 | 658.26 | 0.69 | gga |
| apla-miR-9-5p | 395.66 | 561.49 | 0.71 | gga |
| apla-miR-10a-5p | 7505.07 | 10648.70 | 0.71 | gga |
| apla-miR-128-3p | 1200.80 | 1673.55 | 0.72 | gga |
| apla-miR-1a-3p | 3773.07 | 5208.21 | 0.72 | gga |
| apla-miR-181a-5p | 469.04 | 644.54 | 0.73 | gga |
| novel_31 | 510.87 | 696.45 | 0.73 | gga |
| apla-miR-125b-5p | 878.34 | 1184.91 | 0.74 | gga |
| apla-let-7k-5p | 522.63 | 692.93 | 0.75 | gga |
| apla-miR-26a-5p | 7365.03 | 9520.45 | 0.77 | gga |
| novel_30 | 552.88 | 707.71 | 0.78 | gga |
| apla-miR-100-5p | 5167.32 | 6604.98 | 0.78 | gga |
| apla-miR-199-5p | 2391.52 | 3042.16 | 0.79 | gga |
| apla-miR-199-3p | 2075.97 | 2500.03 | 0.83 | gga |
| apla-miR-21-5p | 65275.81 | 77808.07 | 0.84 | gga |
| apla-miR-146c-5p | 7077.48 | 8411.90 | 0.84 | gga |
| apla-let-7i | 10782.95 | 11944.81 | 0.90 | gga |
| apla-let-7f-5p | 13484.25 | 13116.52 | 1.03 | gga |
| apla-miR-215-5p | 428426.80 | 411990.55 | 1.04 | gga |
| apla-miR-30c-5p | 3376.10 | 3075.59 | 1.10 | gga |
| apla-miR-148a-3p | 133576.70 | 116148.51 | 1.15 | gga |
| apla-miR-92-3p | 3171.27 | 2594.17 | 1.22 | gga |
| apla-miR-30a-5p | 8549.77 | 6964.82 | 1.23 | gga |
| apla-miR-101-3p | 3241.29 | 2613.87 | 1.24 | gga |
| apla-miR-429-3p | 642.69 | 473.33 | 1.36 | gga |
| apla-miR-7 | 10893.68 | 7977.11 | 1.37 | gga |
| apla-miR-194 | 49329.84 | 33719.58 | 1.46 | gga |
| apla-miR-200a-3p | 10564.68 | 6960.59 | 1.52 | gga |
| apla-miR-30e-5p | 1419.27 | 908.48 | 1.56 | gga |
| apla-miR-200b-3p | 11765.48 | 6833.73 | 1.72 | gga |
| apla-miR-375 | 3312.62 | 1224.68 | 2.71 | gga |
| apla-miR-216b | 490.89 | 16.89 | 29.06 | gga |
| apla-miR-217-5p | 1964.87 | 38.18 | 51.46 | gga |

The differentially expressed mRNAs were identified based on RPKM > 100.00 in either of the two groups and P < 0.01. gga, Gallus gallus.
