# Supplementary material for: Analysis of miRNAs and their target genes associated with mucosal damage caused by transport stress in the mallard duck intestine
Source: PLoS One. 2020 Aug 18;15(8):e0237699. doi: 10.1371/journal.pone.0237699 (PMC7437463; doi:10.1371/journal.pone.0237699)
Supplement: S3 Table — (DOCX) [file pone.0237699.s003.docx]

**Table S3. Detailed information of primers.**

| Name | Primer Sequence |
| --- | --- |
| miR-7-PF | CTGGTAGGTGGAAGACTAGTGATTT |
| miR-7-LOOP | CTCAACTGGTGTCGTGGAGTCGGCAATTCAGTTGAGCAACAAA |
| miR-218-5p-PF | CTGGTAGGTACTGCATCAGGAACTG |
| miR-218-5p-LOOP | CTCAACTGGTGTCGTGGAGTCGGCAATTCAGTTGAGATCCAATC |
| miR-101-3p-PF | CTGGTAGGGTACAGTACTGTGATAA |
| miR-101-3p-LOOP | CTCAACTGGTGTCGTGGAGTCGGCAATTCAGTTGAGTTCAGTTA |
| miR-215-5p-PF | CTGGTAGGATGACCTATGAATTGAC |
| miR-215-5p-LOOP | CTCAACTGGTGTCGTGGAGTCGGCAATTCAGTTGAGGTCTGTC |
| miR-217-5p-PF | CTGGTAGGTACTGCATCAGGAACTG |
| miR-217-5p-LOOP | CTCAACTGGTGTCGTGGAGTCGGCAATTCAGTTGAGATCCAATC |
| All-miR-PR | TCAACTGGTGTCGTGGAGTCGGC |
| miR-U6-PF | CTCGCTTCGGCAGCACA |
| miR-U6-PR | AACGCTTCACGAATTTGCGT |
| miR-U6-LOOP | GTCGTATCCAGTGCAGGGTCCGAGGTATTCGCACTGGATACGACAAAAATATG |
| RHOU-PF | GTCATCTCCCACCTACGG |
| RHOU-PR | AACCCACTTCTCACTCACG |
| AKR7A2-PF | TGGGTGATGCCAACTGTCTA |
| AKR7A2-PR | GTGGGTTTGCGAGTGTCTTT |
| EEF1A1-PF | CACCGAGCCACCTTACAG |
| EEF1A1-PR | TGAAGAGGCAGACGCAGA |
| HTRA3-PF | TTGTTACTGGGTCATTCTGC |
| HTRA3-PR | ATTCCAATCACTTCACCATC |
| CHRDL1-PF | AAGCACGGACGAGTTTG |
| CHRDL1-PR | CTCCACGATTCCAAAGG |
| β-actin-PF | ATGTCGCCCTGGATTTCG |
| β-actin-PR | CACAGGACTCCATACCCAAGAA |
