# Supplementary material for: Analysis of miRNAs and their target genes associated with mucosal damage caused by transport stress in the mallard duck intestine
Source: PLoS One. 2020 Aug 18;15(8):e0237699. doi: 10.1371/journal.pone.0237699 (PMC7437463; doi:10.1371/journal.pone.0237699)
Supplement: S1 File — (DOCX) [file pone.0237699.s004.docx]

This work was supported by grants from the National Natural Science Foundation (grant numbers: 31702157), the Hubei Provincial scientific and technological innovation special project (Grant Numbers 2017ABA140) and China Agriculture Research System (Grant Numbers CARS-42-47) and Hubei Academy of Agricultural Sciences Younger Top-Notch Talent Program (Grant Numbers Q2018021).
